# MNL-Network: A Multi-Scale Non-local Network for Epilepsy Detection From EEG Signals

**DOI:** 10.3389/fnins.2020.00870

**Published:** 2020-11-17

**Authors:** Guokai Zhang, Le Yang, Boyang Li, Yiwen Lu, Qinyuan Liu, Wei Zhao, Tianhe Ren, Junsheng Zhou, Shui-Hua Wang, Wenliang Che

**Affiliations:** ^1^School of Optical-Electrical and Computer Engineering, University of Shanghai for Science and Technology, Shanghai, China; ^2^School of Software Engineering, Tongji University, Shanghai, China; ^3^Department of Computer Science and Technology, Tongji University, Shanghai, China; ^4^Chengyi University College, Jimei University, Xiamen, China; ^5^School of Informatics, Xiamen University, Xiamen, China; ^6^School of Architecture Building and Civil Engineering, Loughborough University, Loughborough, United Kingdom; ^7^School of Mathematics and Actuarial Science, University of Leicester, Leicester, United Kingdom; ^8^Department of Cardiology, Shanghai Tenth People's Hospital, Tongji University School of Medicine, Shanghai, China

**Keywords:** convolution neural network, EEG, epilepsy, multi-scale, non-local, seizure, ictal, interictal

## Abstract

Epilepsy is a prevalent neurological disorder that threatens human health in the world. The most commonly used method to detect epilepsy is using the electroencephalogram (EEG). However, epilepsy detection from the EEG is time-consuming and error-prone work because of the varying levels of experience we find in physicians. To tackle this challenge, in this paper, we propose a multi-scale non-local (MNL) network to achieve automatic EEG signal detection. Our MNL-Network is based on 1D convolution neural network involving two specific layers to improve the classification performance. One layer is named the signal pooling layer which incorporates three different sizes of 1D max-pooling layers to learn the multi-scale features from the EEG signal. The other one is called a multi-scale non-local layer, which calculates the correlation of different multi-scale extracted features and outputs the correlative encoded features to further enhance the classification performance. To evaluate the effectiveness of our model, we conduct experiments on the Bonn dataset. The experimental results demonstrate that our MNL-Network could achieve competitive results in the EEG classification task.

## 1. Introduction

As the center of cognitive processes and sensory stimuli, the brain controls the vital functions of the body and has a complicated information processing function (Türk and Özerdem, 2019). When the nervous system is active, the brain emits biopotential signals that can reflect dysfunction or disease. By magnifying and recording the spontaneous biological potential of the brain from the scalp through sophisticated electronic instruments, one can obtain electroencephalography (EEG) signals. Due to its excellent temporal resolution, easy implementation, and low cost, EEG has become one of the most effective techniques in monitoring the brain activity and diagnosing the neurological disorder (Ullah et al., 2018).

Epilepsy is a neurological disorder affecting about 50 million people around the world (Beghi et al., 2005; Megiddo et al., 2016). Epilepsy manifests in the form of seizures, which is an abnormal electrical activity that occurs temporarily in nerve cells (Bancaud, 1973). Since EEG can accurately record the intermittent slow waves, spikes, or irregular spikes during seizures by analyzing the wave morphology of EEG signals, one can give an explicit evaluation of the presence and level of epilepsy. Unfortunately, the detection of epilepsy from EEG requires signal records over a long-term period, which is a time-consuming and inefficient undertaking. Considering the shortage of professional doctors at present, it is therefore urgent and meaningful to detect epilepsy in an automatic way.

In recent years, the algorithms based on the hand-crafted feature engineering have shown great success in many medical image analysis fields (Jiang et al., 2017; Li et al., 2019; Xu et al., 2020); for EEG signal automatic detection tasks, some early attempts such as Gotman (1982) decomposed the EEG into elementary waves and detected the paroxysmal bursts of rhythmic activity. Furthermore, these works could detect the patterns specific to newborns and then give a warning to patients when a seizure is starting (Gotman, 1999). Recently, Gardner et al. proposed a Support Vector Machine (SVM)-based method in which seizure activity induced distributional changes in feature space that increased the empirical outlier fraction (Gardner et al., 2006). Moreover, an automatic epileptic seizure detection method was developed based on line length feature and artificial neural networks in Guo et al. (2010). After that, a different feature acquisition and classification technique in the diagnosis of both epilepsy and autism spectrum disorder (ASD) was developed by Ibrahim (Ibrahim et al., 2017). Lu's team used Kraskov Entropy based on the Hilbert Huang Transform (HHT) to obtain features. They used the Least Squares Version of Support Vector Machine (LS-SVM) for wavelet transformation (Lu et al., 2018). Although many hand-crafted feature algorithms have been proposed, it is still a challenging problem to identify epilepsy and non-epileptic EEG signals due to the noise and artifacts in the data as well as the inconsistency in seizure morphology of the epilepsy (Tao et al., 2017).

Recently, with the great success of deep learning in computer vision and data mining fields, considerable attention has been focused on the EEG signal classification task. Compared with the hand-crafted feature learning methods, the deep learning methods could generically learn stronger discriminative features with an end-to-end manner. For EEG classification, a Computer-Aided Diagnosis (CAD) system was developed in Acharya et al. (2017), which employed the Convolutional Neural Network (CNN) for analysis of EEG signals. In the follow-up study, the authors in Yuan et al. (2017) transformed EEG signals into EEG scalogram sequences using wavelet transformation, and they then obtained three different EEG features by using Global Principal Component Analysis (GPCA) (Vidal, 2016), Stacked Denoising Autoencoders (SDAE) (Vincent et al., 2010), and EEG segments. After that, the seizure detection was performed by combining all the obtained features and assigning them to the SVM classifier. As for the end-to-end feature learning, Türk et al. obtained two-dimensional (2D) frequency-time scalograms by applying continuous wavelet transform to EEG records, and they then used CNN to learn the properties of these scalogram images to classify the EEG signal (Türk and Özerdem, 2019).

Bhattacharyya et al. analyzed the underlying complexity and nonlinearity of EEG signals by computing a novel multi-scale entropy measure for the classification of seizure, seizure-free, and normal EEG signals (Abhijit et al., 2017). Hussein et al. transformed EEG data into a series of non-overlapping segments to reveal the correlation between consecutive data samples. The Long Short Term Memory (LSTM) network and the softmax classifier were exploited for classification to learn the high-level features of normal and seizure EEG models (Hussein et al., 2018). It should be noted that the majority of the automatic systems perform well in detecting binary epilepsy scenarios, but their performance degrades greatly in classifying the ternary case. To overcome this problem, Ullah et al. proposed an ensemble of pyramidal one-dimensional convolutional neural network (P-1D-CNN) models (Ullah et al., 2018), which could efficiently handle the small available data challenge in classifying the ternary case.

Despite some preliminary results that have been established in the literature, they ignore the multi-scale features which play an important role in the EEG classification task. For example, the long scale of the signal reflects more global representations of the EEG signal, and the short scale of signal embodies the information from the local EEG signal. Thus, those methods based on the single scale of the EEG signal could hinder the model from achieving a better performance due to the absence of multi-scale features. Moreover, the correlations of multi-scale signal features could also be an important factor in this classification task. The learned correlations of multi-scale features are capable of providing correlative dependencies of various lengths' EEG signals, which give more feature information to further improve the classification performance. Based on the discussion above, in this paper, we propose a Multi-scale Non-local (MNL) network to learn multi-scale and correlative features from the input EEG signals. The overview of our designed MNL-Network is illustrated in **Figure 2**. Different from the previous work that directly input the extracted features into the fully connected layer for classification, our MNL-Network developed a signal pooling layer to learn the multi-scale representations through different sizes of 1D max-pooling layers (Zhao et al., 2017) and then input these representations to a Non-local layer (Wang et al., 2018), which aims to encode more correlative features with multi-scale characteristic. To evaluate the performance of the proposed MNL-Network, we conduct comprehensive experiments on the public EEG Bonn dataset. The experimental results show the high classification accuracy of different EEG records, which convinces the effectiveness of our MNL-Network.

In the following section, we will first describe the experimental data in section 2.1. The detailed description of our proposed method is introduced from section 2.2 to section 2.4. The comparison results of different class combinations will be presented in section 3. Finally, we will give an overall discussion of our work in section 4.

## 2. Data and Methods

### 2.1. Data Description

**Figure 1:**
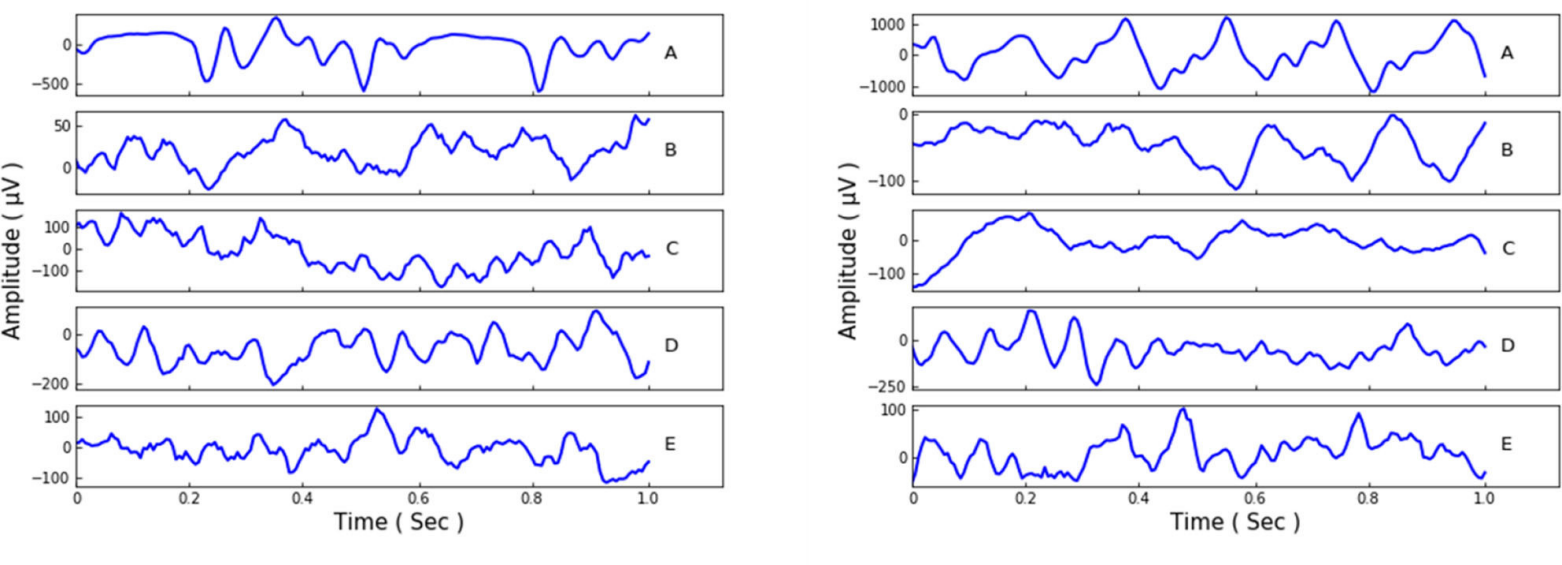
The data samples from the Bonn dataset.

Our experiments employ the EEG Bonn dataset (Andrzejak et al., 2001), which is public and widely used. There are five subsets/classes in this Bonn dataset, and they are denoted as set A, B, C, D, and E. Set A and B monitor the surface EEG records of healthy waking people with eyes open or closed. The other three sets, C, D, and E, are collected from epileptics. Set C it detects the records from the hippocampal formation of the brain during the seizure-free intervals. Set D is gained from the epileptogenic zone with the same intervals as set C, and set E only contains the records of the seizure activities. Each of the five sets is composed of 100 person signals of sampling rate 173.61 Hz and duration 23.6 s. Afterwards, the data samples were made into 4, 097 data points and then divided into 23 chunks for each person signal. Thus, the total record amount of the five sets could be 23 × 100 × 5 = 11, 500, and each set contains 2, 300 records. We show some samples of the different sets in Figure 1.

### 2.2. Network Architecture

**Figure 2:**
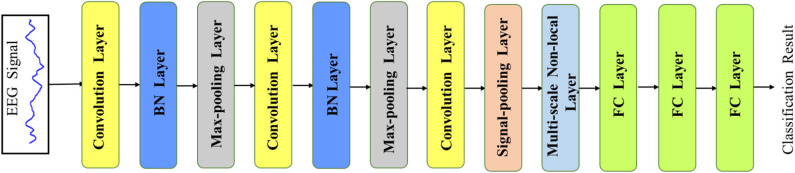
The overview of our MNL-Network. The main backbone is based on a 1D convolution neural network with two additional layers (i.e., signal pooling layer and multi-scale non-local layer), which are developed to learn multi-scale and correlative features from the EEG signals.

Recently, the deep convolutional neural network has achieved great success in computer vision and data analysis fields, and it has become the most rapidly developing technology in the machine learning domain. Compared with the traditional hand-defined feature learning methods, the CNN extracts highly sophisticated feature representations by an end-to-end learning mode, which could be more efficient and accurate. Since the EEG signal is a 1D time-series data, our main network is based on a 1D CNN, which mainly consists of the convolution layer, max-pooling layer, batch-normalization (BN) layer, and fully connected (FC) layer. The overview of our proposed network is illustrated in Figure 2. The network takes the EEG signal as the input and outputs the final EEG classification prediction result in an end-to-end manner. In order to accelerate the convergence of the network, we first use the z-score normalization to normalize the input EEG signal to [0, 1] range. Denoting the input signal data as *s*, the z-score normalization could be formulated as the following:

(1) $$\begin{array}{l} s^{*}=\frac{s-\mu }{\theta } \end{array}$$

where μ is the mean value of *s*, the parameter of θ is the standard deviation of *s*, and the normalized data is s^*^.

In our MNL-Network, the first layer is the convolution layer, which is generally used for filtering signals with fixed length to get discriminating features from the input. The filter number denotes the amounts of features that the kernel extracts. For reducing the complexities of the network, we use three convolution layers with the sizes 40, 20, and 10, respectively. Through using different kernels, discriminative categories of features are extracted and then fed into the next layer. Note that in the CNN, the deeper convolution layer usually extracts more high-level representations, while the lower one learns more tiny-detailed features. For learning more non-linear representations from the EEG signal, the Rectified Linear Unit (RELU) activation function is adopted with the form as follows:

(2) $$\begin{array}{l} f\left(a\right)=max\left(0,a\right) \end{array}$$

where *f*(*a*) is the activation output of the input feature *a*. Since the BN layer can accelerate the learning process and maintain training stability, we add it after each convolution layer. After the BN layer, the max-pooling layer is followed to get the maximum signal value from the encoded features, and it is also used to down-sample or pool the input representation. The size of the max-pooling layer in our network is set as 2 with the stride 2. Before input the extracted features into the signal pooling layer, there are two max-pooling layers utilized to extract spatial information and enlarge the receptive field from the signal features. The detailed parameters of the MNL-Network are presented in Table 1.

**Table 1:** The parameters of the MNL-Network.

**Layer**	**Filter numbers**	**Filter size**	**Stride**	**Output size**
Convolution layer	20	40	–	178 × 20
BN layer	–	–	–	178 × 20
Max-pooling layer	–	2	2	89 × 20
Convolution layer	40	20	2	35 × 40
BN layer	–	–	–	35 × 40
Max-pooling layer	–	2	2	17 × 40
Convolution layer	80	10	2	4 × 80
BN layer	–	–	–	4 × 80
Signal pooling layer	–	–	–	4 × 80
Multi-scale non-local layer	–	–	–	4 × 80
Flatten	–	–	–	4 × 80
FC layer	64	–	–	64
FC layer	32	–	–	32
FC layer	2	–	–	5

For combining non-linear features from the previous layers, we use three FC layers, and the last FC layer is with a softmax function to output the prediction probability of each class. Mathematically, we denote the class labels as *y*_(*i*)_ ∈ {1, 2, ⋯ , *C*}, where the data samples have *C* classes totally. Given the normalized input data s^*^, the softmax operation $h_{\theta }({\text{s}}^{*})$ could be formulated as the following:

(3) $$\begin{array}{l} \;h_{\theta }({\text{s}}^{*})=\left(\begin{array}{c} P(y=1|{\text{s}}^{*};\theta )\\ P(y=2|{\text{s}}^{*};\theta )\\ \vdots \\ P(y=C|{\text{s}}^{*};\theta ) \end{array}\right)\\ =\frac{1}{\sum_{j=1}^{C}\exp\left(\theta_{j}^{T}{\text{s}}^{*}\right)}\left(\begin{array}{c} \exp\left(\theta_{1}^{T}{\text{s}}^{*}\right)\\ \exp\left(\theta_{2}^{T}{\text{s}}^{*}\right)\\ \vdots \\ \exp\left(\theta_{C}^{T}{\text{s}}^{*}\right) \end{array}\right) \end{array}$$

where θ_1_, θ_2_, ⋯ , θ_*C*_ are the parameters of the softmax operation.

### 2.3. Signal Pooling Layer

**Figure 3:**
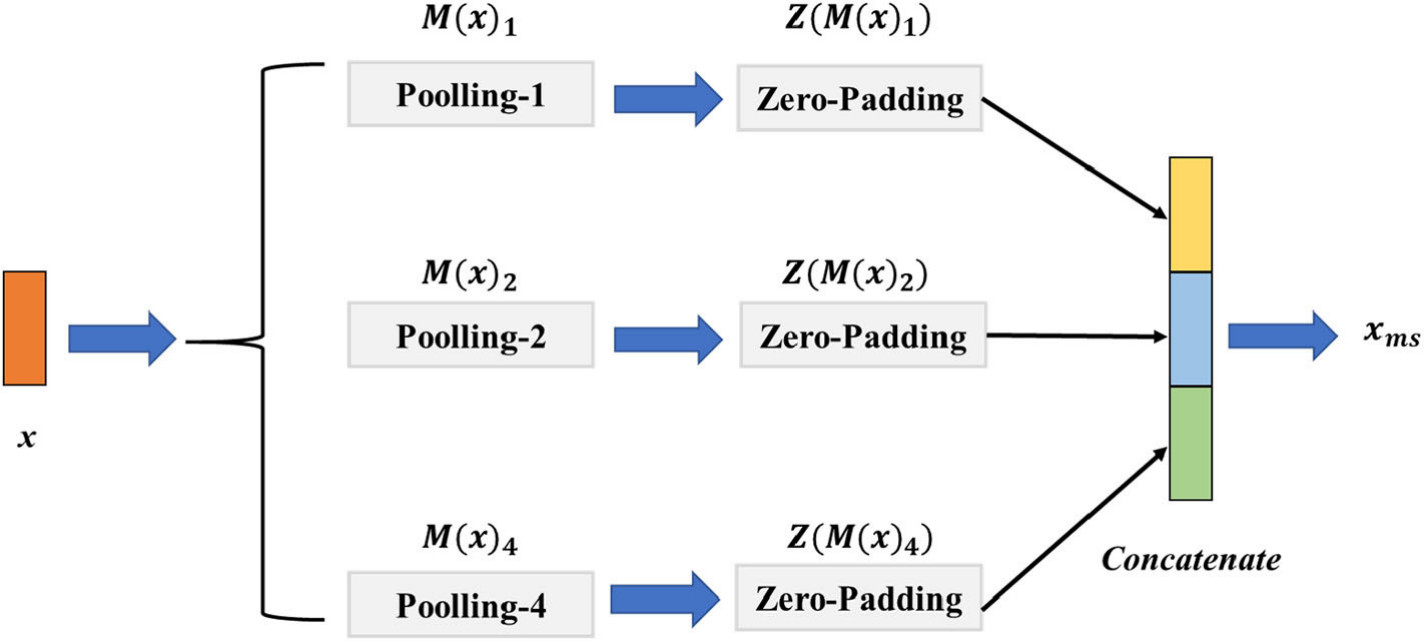
The architecture of the signal pooling layer. The *M*(*x*)_1_, *M*(*x*)_2_, *M*(*x*)_4_ denote the max-pooling operations with the sizes of {1, 2, 4}, respectively, and the *Z*(*M*(*x*)_1_), *Z*(*M*(*x*)_2_), *Z*(*M*(*x*)_4_) is the zero-padding layer with sizes of {1, 2, 4} that aim to reshape the feature map resolution to the same size.

The EEG signal with different scales contains various multi-scale representations. However, the fixed size of the convolution or pooling layer could ignore the multi-scale features and thus hinder the model from achieving a higher classification performance. To address this challenge, we introduce a signal pooling layer that has two main parts: the multi-pooling part and the concatenation part to realize extracting the features from different scales. The detailed structure of the signal pooling layer is illustrated in Figure 3. Let **x** ∈ ℝ^*w*×*c*^ be the output features from the third convolution layer, *w* the length of **x**, and *c* indicate the channels of **x**. Then, we define the **M**(·)_*p*_ as the max-pooling operation with the size of *p* ∈ {1, 2, 4} and the stride as *d* with the value of 1; the output size of feature *o* could be calculated as Equation (4).

(4) $$\begin{array}{l} o=\left(w-p\right)/d+1 \end{array}$$

For merging different sizes of the multi-scale features, we define the operator of **Z**(·) is the zero-padding layer with the left and right padding size of *l* and *r*, and the value of *l*, *r* is calculated as follows:

(5) $$\begin{array}{l} l=\left\lceil \frac{w-o}{4}\right\rceil \end{array}$$

(6) $$\begin{array}{l} r=w-o-l \end{array}$$

where ⌈·⌉ denotes the round up value operation. Specifically, for the input feature **x**, we first perform **M**(·)_1_, **M**(·)_2_, **M**(·)_4_ parallelly to extract the multi-scale features. We then use the **Z**(·) to pad the features to the same size. Finally, a concatenation operation of **Z**(**M**(**x**)_**1**_), **Z**(**M**(**x**)_**2**_), **Z**(**M**(**x**)_**4**_) is conducted before taking them into the multi-scale non-local layer. The final output feature **x**_*ms*_ of the signal pooling layer could be given as follows:

(7) $$x_{ms}=Concat(Z(M{\left(x\right)}_{1}),Z(M{\left(x\right)}_{2}),Z(M{\left(x\right)}_{4}))$$

where the *Concat*(·) represents the concatenation operation of different features.

### 2.4. Multi-Scale Non-local Layer

**Figure 4:**
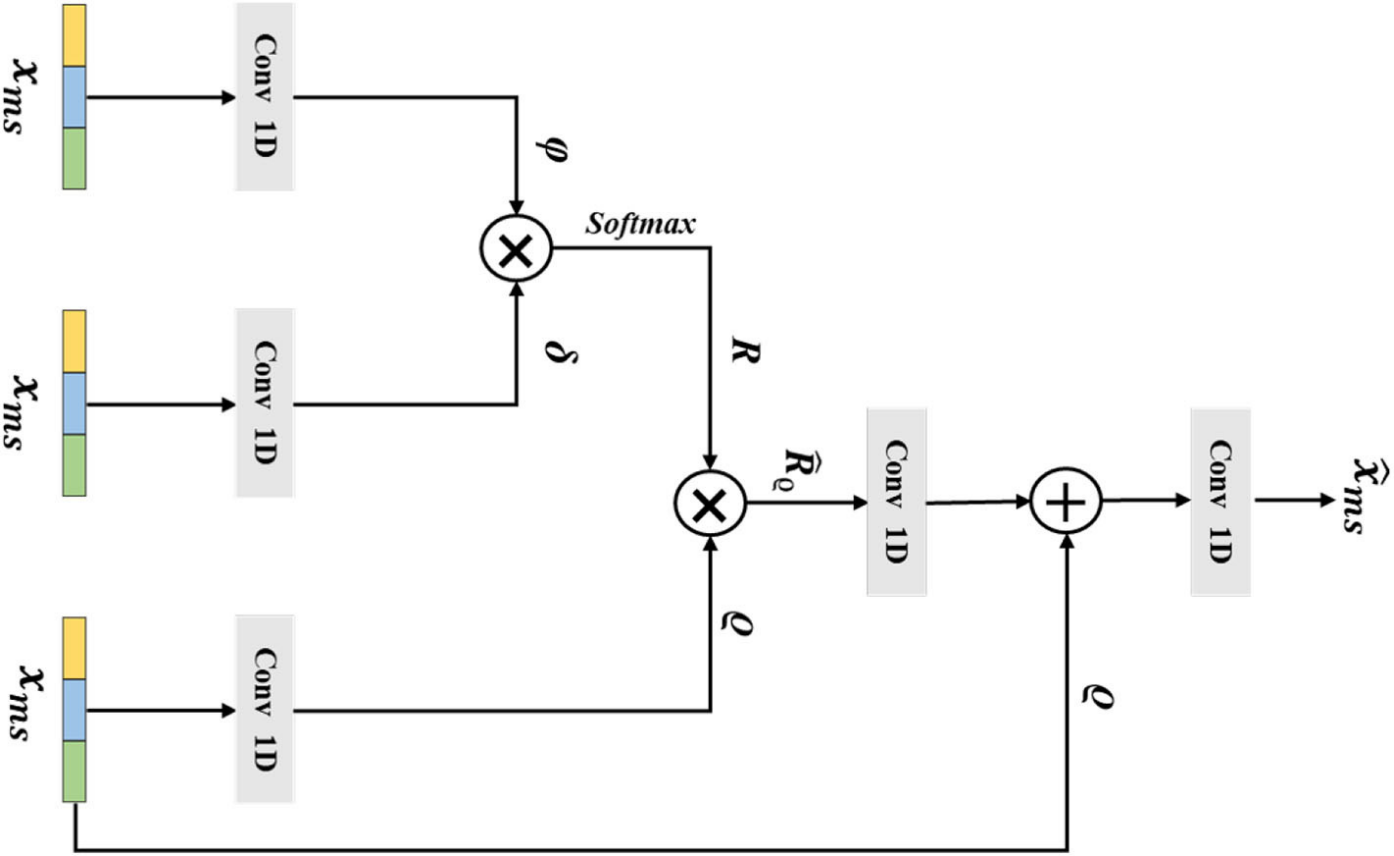
The architecture of the multi-scale non-local layer. The **x**_*ms*_ is the extracted multi-scale features from the previous layer, ϕ, δ, *and* ϱ are the output features from Conv 1D, the variable of **R** denotes the similarity matrix of ϕ and δ, the ${\hat{\text{R}}}_{\varrho }$ is gained by multiplying **R** and ϱ, and the final output of the multi-scale non-local layer is ${\hat{\text{x}}}_{ms}$.

The long-range correlations of different scale EEG signals are of a vital importance in the epilepsy classification task. However, the traditional non-local method has been impeded by the lack of considering the multi-scale features. Thus, in this section, we introduce our designed multi-scale non-local layer, which could learn discriminative multi-scale EEG signal features in a non-local manner. The detailed structure of our multi-scale non-local layer is shown in Figure 4. Instead of using the hierarchical feature from the network, the input feature of the multi-scale non-local layer is extracted from the signal pooling layer, which contains more discriminative multi-scale representations. By measuring the correlations of different multi-scale features, the final category of the EEG signal could be predicted by learning similarities across different scopes.

Mathematically, we consider that the multi-scale input feature of the multi-scale non-local layer is ${\text{x}}_{ms}\in {\mathbb{R}}^{w\times c}$, which is extracted from the previous signal pooling layer. Then, we use three 1D convolutions with the receptive field and filter size of one to transform ${\text{x}}_{ms}\in {\mathbb{R}}^{w\times c}$ to embedding space, the output features ϕ ∈ ℝ^*w*×ĉ^, δ ∈ ℝ^*w*×ĉ^, ϱ ∈ ℝ^*w*×ĉ^ could be defined as:

(8) $$\begin{array}{l} \phi =Conv_{\phi }\left({\text{x}}_{ms}\right) \end{array}$$

(9) $$\begin{array}{l} \delta =Conv_{\delta }\left({\text{x}}_{ms}\right) \end{array}$$

(10) $$\begin{array}{l} \varrho =Conv_{\varrho }\left({\text{x}}_{ms}\right) \end{array}$$

where ĉ is the channel number of ϕ, δ, *and* ϱ. After that we flatten the three embeddings and use the matrix multiplication operation *g*(·, ·) between ϕ and δ to calculate the similarity matrix **R**, which could be formulated as the following:

(11) $$\begin{array}{l} \text{R}=g\left(\phi ,\delta \right)=\phi^{\text{T}}\times \delta \end{array}$$

Next, we apply the a softmax operation to *h*_η_(·) to normalize the similarity matrix **R** and gain the attention weight matrix $\hat{\text{R}}$ =*h*_η_(**R**); here, η is the parameters of softmax operation. Then, we perform a matrix multiplication between $\hat{\text{R}}$ and ϱ, which is formulated as the following:

(12) $$\begin{array}{l} {\hat{\text{R}}}_{\varrho }=\hat{\text{R}}\times \varrho^{\text{T}} \end{array}$$

and the ${\hat{\text{R}}}_{\varrho }\in {\mathbb{R}}^{w\times ĉ}$ denotes the output feature. Afterward, a residual connection between $\hat{\text{R}}$ and ϱ is performed, and the final output ${\hat{\text{x}}}_{ms}$ of the multi-scale non-local layer is given as the following:

(13) $$\begin{array}{l} {\hat{\text{x}}}_{ms}=Conv_{ms}\left(Conv_{ms}\left(\hat{{\text{R}}_{\varrho }}\right)+{\text{x}}_{ms}\right) \end{array}$$

### 2.5. Implementation Details

The proposed method is implemented by the Keras with 1 RTX 2070 GPU, and we use the cross-entropy as our loss function to train the model end-to-end. The parameters of the method are optimized by the Adam optimizer, the initial learning rate is set as 0.0005, and we reduce it by 0.1 after the validation accuracy is not improved after 10 epochs. All the training data is trained in a mini-batch size mode, and we set the mini-batch as 100 for each epoch. For each fold, we choose the best checkpoint on the validation accuracy as our final predicted model.

## 3. Experimental Results

### 3.1. Evaluation Metrics

For evaluation, well-known performance metrics, such as accuracy, precision, sensitivity, specificity and F1-score, are adopted. The definitions of these performance metrics are given below:

(14) $$\begin{array}{l} Accuracy=\frac{TP+TN}{TP+TN+FP+FN} \end{array}$$

(15) $$\begin{array}{l} Precision=\frac{TP}{TP+FP} \end{array}$$

(16) $$\begin{array}{l} Sensitivity=\frac{TP}{TP+FN} \end{array}$$

(17) $$\begin{array}{l} Specificity=\frac{TN}{FP+TN} \end{array}$$

(18) $$\begin{array}{l} F1-score=2\times \frac{Precision\times Sensitivity}{Precision+Sensitivity} \end{array}$$

where TP (true positives) are the number of the EEG records that are abnormal and actually identified as abnormal; TN (true negatives) are the number of the EEG records that are normal and actually identified as normal; FP (false positives) are the number of the EEG records that are normal but are actually predicted as abnormal; and FN (false negatives) are the number of the EEG records that are abnormal but are actually predicted as normal.

In order to ensure the system is tested over different categories of data, we used 10-fold cross-validation to evaluate all the data in system performance. That is, we randomly divided the 2,300 EEG signals of each class into ten non-overlapping folds. Then each fold, in turn, is used for testing while the other nine folds are used for training the model. We calculated the average values of accuracy, sensitivity, and specificity for 10-folds to get the average performance of the system.

### 3.2. The Performance of Double Classes Classification

In this section, we conduct the experiment by comparing the double classes classification performance. The combinations of different classes are A-E, B-E, C-E, D-E, AB-E, AC-E, AD-E, BC-E, BD-E, CD-E, ABC-E, ABD-E, BCD-E, and ABCD-E. The accuracy comparison result of k-10 testing is shown in Table 2, the best performance is achieved on the A-E classes classification with the performance of 99.93%, and the hardest classification is CD-E with the score of 98.54%. It could explain that the healthy waking with eyes open classes could have a big difference from the seizure epileptic, and thus it could achieve a higher classification performance. We also compare other metrics as shown in Table 3. The best overall performance is by classifying A-E classes, which further proves the reasons presented above. Overall, the accuracy performance of different double classes is all above 98%, which indicates that our proposed method could be very generalized in this classification task.

**Table 2:** The k-10 accuracy performance of double classes classification.

	**k1**	**k2**	**k3**	**k4**	**k5**	**k6**	**k7**	**k8**	**k9**	**k10**	**Mean**
A-E	97.82	100	100	99.78	100	99.78	100	100	100	100	99.93
B-E	99.35	100	100	99.35	99.78	99.57	99.78	100	99.35	100	99.72
C-E	98.45	99.57	99.13	99.78	99.78	99.56	99.13	98.91	99.13	100	99.35
D-E	98.70	98.48	99.57	98.91	98.48	99.35	98.70	98.70	98.70	98.04	98.76
AB-E	99.57	100	99.86	99.42	99.71	99.71	99.71	100	99.57	100	99.75
AC-E	99.42	99.42	99.57	99.57	99.42	99.71	99.28	99.28	99.28	99.86	99.48
AD-E	98.99	99.42	99.13	99.28	98.84	98.99	98.70	99.28	98.99	99.42	99.10
BC-E	99.28	99.86	99.42	99.28	98.99	99.28	99.13	99.57	98.99	100	99.38
BD-E	98.84	98.84	98.99	98.84	98.26	98.99	98.99	98.99	98.16	99.57	98.84
CD-E	98.84	99.42	98.99	99.28	98.12	98.99	99.70	98.12	98.70	99.28	98.84
ABC-E	99.57	99.67	99.78	99.24	99.78	99.67	99.57	99.78	99.57	99.24	99.59
ABD-E	99.46	99.13	99.24	99.02	99.57	99.78	99.24	99.57	99.13	99.13	99.33
BCD-E	99.57	98.70	99.24	98.70	99.35	99.35	98.59	99.35	98.91	98.04	98.98
ABCD-E	99.04	99.39	99.39	99.13	98.87	99.48	99.48	98.70	99.48	98.70	99.17

**Table 3:** The overall performance of double classes classification.

	**Accuracy**	**Sensitivity**	**Specificity**	**Precision**	**F1-score**
A-E	99.93	98.96	99.96	99.96	99.45
B-E	99.72	97.96	99.91	99.91	98.92
C-E	99.35	96.70	99.83	99.82	98.23
D-E	98.76	97.09	98.30	98.31	97.68
AB-E	99.75	97.87	99.98	99.96	98.90
AC-E	99.48	96.91	99.85	99.69	98.28
AD-E	99.10	97.04	99.48	98.94	97.98
BC-E	99.38	96.61	99.89	99.78	98.16
BD-E	98.84	95.70	99.48	98.93	97.28
CD-E	98.84	95.30	99.61	99.18	97.20
ABC-E	99.59	96.13	99.97	99.91	97.98
ABD-E	99.33	96.57	99.58	98.72	97.62
BCD-E	98.98	94.39	99.62	98.83	96.53
ABCD-E	99.17	94.65	99.76	99.00	96.77

### 3.3. The k-10 Performance of Multiple Classes Classification

For a more comprehensive comparison, we conduct multiple classes classification in this section. The experimental combinations contain A-C-E, A-D-E, B-C-E, B-D-E, AB-CD-E, and A-B-C-D-E, separately. The accuracy performance of different multiple classes is illustrated in Table 4. Compared with the double classes classification, the multiple classes classification tends to be more difficult, and the overall accuracy is lower than the double classes. The reason behind this could be the multiple classes classification having a more complex data distribution than the double classes classification. The overall comparison result of other metrics is shown in Table 5. The best performance is gained by the B-D-E combination, and it achieves 98.62% accuracy, 97.14% sensitivity, 98.57% specificity, 97.14% precision, and an F1-score of 97.14%. Meanwhile, the result shows that the A-B-C-D-E five classes combination obtains the lowest performance, and it is mostly because the five classes combination has a more complicated data characteristic from each class data.

**Table 4:** The k-10 performance of multiple classes classification.

	**k1**	**k2**	**k3**	**k4**	**k5**	**k6**	**k7**	**k8**	**k9**	**k10**	**Mean**
A-C-E	97.34	97.78	96.81	97.68	97.00	98.12	98.16	98.02	97.20	97.68	97.58
A-D-E	97.68	97.83	98.16	97.83	97.49	97.87	98.36	97.97	97.54	97.39	97.81
B-C-E	98.07	97.83	98.60	97.78	98.16	98.60	98.84	99.42	97.97	99.03	98.43
B-D-E	98.45	98.74	98.79	98.31	98.74	98.36	98.65	98.74	98.55	98.84	98.62
AB-CD-E	97.04	97.39	98.67	97.62	97.68	97.91	97.62	97.97	98.55	97.13	97.76
A-B-C-D-E	94.63	94.35	93.34	93.79	93.98	94.40	94.28	93.11	93.93	94.31	94.01

**Table 5:** The overall performance of multiple classes classification.

	**Accuracy**	**Sensitivity**	**Specificity**	**Precision**	**F1-score**
A-C-E	97.58	95.54	97.77	95.54	95.54
A-D-E	97.81	95.87	97.93	95.87	95.87
B-C-E	98.43	97.00	98.50	97.00	97.00
B-D-E	98.62	97.14	98.57	97.14	97.14
AB-CD-E	97.76	95.95	97.97	95.95	95.95
A-B-C-D-E	94.01	83.49	83.49	95.87	89.46

### 3.4. Compare With Other Methods

To further evaluate the effectiveness of our proposed network, we compare our method with other previous works. In Table 6, it shows the comparison result of different methods. Since there are multiple combinations of EEG classes, for simplicity, we use the A-E, B-E, C-E, D-E, AB-E, BC-E, CD-E, A-D-E, and A-B-C-D-E combinations to evaluate our MNL-Network performance. The comparison result demonstrates that our method could achieve competitive performance on double classed classification when compared with other previous works. The best performance of the double classes combination is achieved by A-E, and the reason could be that the class A and E have a large gap between each other, and some other combinations also gain high classification accuracy, which are all above 94%. Moreover, the five classes classification result is also reported in Table 6, our proposed method has achieved an accuracy classification performance of 94.01%, which is higher than the recent works. In particular, the overall CNN based methods usually have better performance than the traditional hand-crafted ones, which further proves that they can extract stronger discriminative representations thus could perform more prominently in the classification task.

**Table 6:** The overall performance of double classes classification.

	**Method**	**Study**	**Accuracy**	**Our accuracy**
	1-D-LBP + FT/BN	Kaya et al., 2014	99.50	
	FFT and Decision tree	Polat and Güneş, 2007	98.70	
A-E	Wavelet transform	Lee et al., 2014	98.17	99.93
	Artificial neural networks	Nigam and Graupe, 2004	97.50	
	CWT + CNN	Türk and Özerdem, 2019	99.50	
	Robust CNN	Zhao et al., 2020	99.11	
	CNN + M-V	Ullah et al., 2018	99.6	
B-E	CWT + CNN	Türk and Özerdem, 2019	99.50	99.72
	DTCWT + GRNN	Swami et al., 2016	98.9	
	DWT + NB/KNN	Sharmila, 2016	99.25	
	CWT + CNN	Türk and Özerdem, 2019	98.50	
	CCNN + M-V	Ullah et al., 2018	99.1	
C-E	DTCWT + GRNN	Swami et al., 2016	98.7	99.35
	Robust CNN	Zhao et al., 2020	99.1	
	P-1D-CNN	Ullah et al., 2018	98.02	
	TQWT- K-NN Entropy	Abhijit et al., 2017	98.00	
	CEEMDAN + RF	Jia et al., 2017	98.00	
D-E	DTCWT + GRNN	Swami et al., 2016	98.00	98.76
	Robust CNN	Zhao et al., 2020	97.63	
	WPE + SVM	Tawfik et al., 2016	96.50	
	DWT + NB/KNN	Sharmila, 2016	99.16	
AB-E	DTCWT + GRNN	Swami et al., 2016	99.2	99.75
	Robust CNN	Zhao et al., 2020	99.38	
BC-E	DWT+NB/K-NN	Sharmila, 2016	98.3	99.38
	1-D-LBP + FT/BN	Kaya et al., 2014	97.00	
CD-E	DWT + NB/KNN	Sharmila, 2016	98.75	98.84
	Robust CNN	Zhao et al., 2020	98.03	
	1-D-LBP + FT/BN	Kaya et al., 2014	95.67	
A-D-E	LSP-SVM	Tuncer et al., 2019	95.67	97.81
	TQWT-QSP + 1N	Aydemir et al., 2020	99.67	
	LSP-SVM	Tuncer et al., 2019	93.0	
A-B-C-D-E	Robust CNN	Zhao et al., 2020	93.55	94.01

## 4. Conclusion

In this paper, we propose an automatic EEG signal detection network to help the physicians diagnose the epilepsy more efficiently. The whole architecture is based on the 1D convolution neural network, and two additional layers (signal pooling layer and multi-scale non-local layer) are proposed to learn the multi-scale and correlative information. Extensive comparative evaluations on the Bonn dataset are conducted, and they validate the effectiveness of our proposed method. In future works, we will explore the possibilities of incorporating reinforcement learning in this classification task.

## Data Availability Statement

Publicly available datasets were analyzed in this study. This data can be found here: https://archive.ics.uci.edu/ml/datasets/Epileptic+Seizure+Recognition.

## Author Contributions

GZ, LY, and BL conceived the idea and designed the algorithm. GZ conducted the experiments. YL wrote the initial paper. All the remaining authors contributed to refining the ideas and revised the manuscript.

## Conflict of Interest

The authors declare that the research was conducted in the absence of any commercial or financial relationships that could be construed as a potential conflict of interest.
